# The Classes and the Masses: A Word to the Wise

**Published:** 1886-12-18

**Authors:** 


### The Classes and the Masses: A Word to the Wise.

By the Ven. Archdeacon Farrar, D.D.

### I.—THE LESSONS OF THE TIMES.

The Effects of a City Life.

The circumstances of this season of the year naturally turn our thoughts to the existing distress, and to the whole question of the relation of poverty to wealth. There never has been an age since the world was, in which there has not been a contrast between the lots of the rich and of the poor; but in southern and eastern lands, where life is easily maintained, poverty is never so pressing as in lands like ours. The life of England is, unfortunately, becoming more and more a city life, and it is in cities, above all in great cities, that the contrast becomes most glaring, and the distress at times so ghastly as to madden the multitude with a sense, however blind, of intolerable wrong. And when this is the case it has often been the sign of social decay, the omen of impending ruin. What was one cause of the downfall of ancient Rome? What was the cause of the French Revolution? It was the hard reality of inexpressible misery brushed by the rustling masquerade of careless luxury. One day, as Louis XV was hunting in the wood of Senart, away from the gorgeous and guilty palace of Versailles, he met a ragged peasant with a coffin. "What did the man die of?" asked the king—"Of hunger," said the serf, and the king gave his steed the spur. When Foulon was asked how the overtaxed people were to live, he brutally answered, "Let them eat grass." When the mob, maddened into wild beasts, caught him in the streets of Paris, they hanged him, and stuck his head on a pike, his mouth filled with grass, amid sounds as of Tophet from a grass-eating people. What is history but a reflection of the experience of the past for the warning of the present? In these days it is the duty, not only of every Christian, but of every patriot, of every lover of his fellow man, to think often and seriously of his duties to the poor. Whether distress is more or less universal now than in past days, is a question which we need not consider; suffice it for our duty, and for our sympathy, that distress there is; and for Christians there can be no sight more solemn or saddening than that of wealth, a monster, gorged amidst starving populations. In a society so complicated as ours the change of a fashion, the shifting of a tax, the accident of a discovery, the change in a line of commerce, may affect the livelihood of thousands.

Not an Exaggerated Picture.

I shall not shrink from quoting words in the pamphlet of a Socialist as to what this means: "It means to gradually sell or pawn the few sticks of furniture which convert the single room into a home; to blister the feet in walking in search of work, while hope deferred makes the heart sick, and want of nourishment enfeebles the frame; to see your wife sinking for lack of food, and to send your children to the Board School without breakfast; to know that as you grow each day more gaunt in face, more shabby in appearance, more emaciated in physique, there is less and less chance of obtaining employment; to return faint and footsore, after a long day's tramp, and hear those whom you love best on earth crying for want of food; to ponder, in cold and hunger, whether the theft which might save your family from starvation is a crime or a duty; to be restrained from suicide only by the certainty that your death will drive your helpless daughters to swell the ghastly army of degraded womanhood; to feel drawing ever nearer to the day when you will be driven alone into the living tomb of the workhouse; to feel through all this that you have done nothing to deserve it—that is what it means to be out of work." And the picture is not exaggerated. I claim your attention, I claim the attention of the nation, to it, and the question at once arises, if there be this deep distress how is it to be remedied?

How Nations have Dealt with Distress.

Let us see how nations and classes sometimes deal with it. Sometimes, and this is the very worst and basest way of all, they treat it with neglect and indifference, shut their eyes hard to it, ignore it altogether. This most fatal course is possible, but not for long. It is possible for a time for men to make colossal fortunes by grinding the faces of the poor, to surround themselves with every form of luxury, to make the calendar of the year one round of careless, heartless, selfish dissipation, to be indifferent to the fermenting mass of unhappy human beings around them, to encourage the traffickers in drink and poison, and lust and death and spurious excitement, until the society beneath them is an accumulation of Dead Sea wreckage. So it was in the France of 1750; the population growing daily more and more wretched, more and more vicious, more and more ferociously sullen, more and more madly discontented, till the low moan and muttering of the ground-swell in this heaving sea of miserable humanity burst forth into the roar of the flood and typhoon. Of all courses which a nation and its rich and its rulers can take, the indifference to social problems, the neglect of social problems, the mere *laissez faire* as to social problems, is the most insensate and most base. Another way of dealing with distress is the sudden adoption of spasmodic, ill-considered, panic-stricken remedies, which only intensify the virulence of the disease. It is like the policy of Ethelred the Unready in buying off the incursions of the Danes.

**A Contemptible but Common Remedy.** One of the worst and commonest of remedies is a remedy altogether temporary and contemptible; it is that of indiscriminate doles, it is the perpetual feeding of a foul disease, it neglects the sufferers to support the rogues. The person who without inquiry gives his money to the hypocritic whine and lying tale of professional beggars, is flinging it away in the encouragement of lazy imposture. Such mercy is not mercy, it is pure selfishness; it is twice cursed, it curses him who gives and him who takes. There are classes of the community whom it is a simple wrong to the community and to themselves to encourage in their worthlessness. The London roughs, the London criminals, the professional pickpockets and burglars, who make life a terror to myriads of unprotected households; the blaspheming groups which loaf about the thievish corners of the streets, bloated by depravity and gin; the wretches who haunt the parks to levy blackmail by trumping up lying charges against the innocent; the brutal bullies who assault helpless girls and snatch purses from helpless women, —these are birds of prey to which every society true to itself should mete out a pitiless justice; and sturdy vagabonds, and begging-letter writers, and rogues who go about with sham deformities or stolen children, are hardly less noxious and depraved. When you give to these you are not giving indiscriminate charity, you are doing indiscriminate mischief. To consider the poor is a high and blessed thing, to fling chance doles to the drunken and the worthless is a mere baseness and folly. Relief funds administered haphazard may only do the same harm on a larger scale. The East End of London, according to some who know it and who know it best, has been irretrievably demoralised by the careless scattering of ill-considered gold. It is only when we give wisely and generously that God will approve our gifts; and the wise giving of money becomes a most stringently obligatory duty in exact proportion as we take no personal part in those forms of kindness which are more personally blessed.

**From Negative to Positive.** If nothing but evil can come from neglected indifference, from indiscriminate almsgiving, from panic-stricken legislation, from Socialistic revolution, are we to sit still and do nothing? God forbid! There is a world of room for Christian effort, there is ample work to be done by every human being who has risen sufficiently high in the scale of human beings to have a brain to think, a heart to pity, or an arm to aid. So far as the Socialists are moved by deep compassion for human misery—as I am persuaded some of them are moved,—so far as their action may serve to startle us from a selfish apathy, so far as they succeed in opening the eyes of this nation to a state of things which will tax all the wisdom of the wise and all the mercy of the kind to remedy, so far they may well have the sympathy of us all, even when we are compelled to consider many of their words inflammatory, and some of their methods as a certain cause of deeper misery and of worse complications. But there is one remedy which goes to the very heart of the matter, and if the Socialist leaders, whom, as I have said, I regard to be, as far as I know them, sincere men, if they desire to benefit and not to madden, to uplift and not to flatter, it is their special duty of all men to make their adherents see that if in this nation distress is to be relieved and pauperism is to be abolished, then they must do their utmost to cut off the most prolific and permanent cause of distress and pauperism. He is a better patriot and a truer philanthropist who cuts off the cause than he who potters with the effects; he who prevents the disease than he who merely alleviates or drives inward the symptoms. Now, it is a very sad fact, which we must confront, that besides the distress which is innocent and undeserved, and which needs all our sympathy and all our efforts, there is a great deal of distress—how much God only knows—which is absolutely self-caused, which is the necessary consequence of laziness and vice. It is a duty to say this. It will not be relished, but truth is seldom relished. If it is a duty to speak of the sins and vices of the rich, it is no less necessary, and in these days when the working-classes are our practical masters, it requires more courage to point out, not harshly, not unsympathetically, yet with perfect faithfulness, the sins and vices of the poor.

**Three Causes of Distress.** Now among the poor there are three widespread and prolific causes of distress—they are *thriftlessness*, *disgracefully-early marriages*, and above all, *drink*. First, there is *thriftlessness*. The poor clergyman, the poor clerk, the poor tradesman is, as a rule, thrifty; he denies himself, he lives within his narrow income, he puts by some of his scanty earnings; in the summer he does not forget winter, nor in sunshine the rainy days. It is not so with multitudes of the poor. When in good wages, many of them waste what would have kept their self-respect when work is slack. They have not realised, as a class, that extravagance and luxury are quite as possible and quite as culpable in the poor as in the rich, and that if it sometimes be extravagance and luxury to spend money on jewels or on flowers, it may be a worse extravagance and a worse luxury to spend money on gin and beer. The second cause of distress is the prevalence of *disgracefully-early marriages*. In the upper and middle classes it is an exception, and it is regarded as discreditable, if a young man thinks of marrying much before the age of twenty-nine or thirty, and unless he has sufficient means to keep his wife and family; but, unhappily, in the poorest classes in parts of London marriages between mere boys and girls of eighteen or nineteen are disgracefully prevalent, and they marry often when they have no more in hand than to pay a month's rent for some filthy and squalid room.

Hence, as statistics prove, the rate of increase of our population, which is so appalling a problem, is very much more rapid in squalid centres than in the wealthier suburbs, and the result—inevitable by those laws which God Himself has impressed on human society—the result inevitably is an offspring stunted, ricketty, diseased, unhappy children of the gutter and the slum, who, on the borderlands of destitution and misery, are, in the terrible language of South, "not so much born into the world as damned into the world." Beyond all question, and beyond all remedy, pauperism will be multiplied, and misery deepened, till the poor learn, as well as the rich, that marriage is not to be enterprised, or taken in hand unadvisedly, lightly, or wantonly, to satisfy men's carnal lusts and appetites, like brute beasts that have no understanding, but discreetly, advisedly, reverently, soberly, and in the fear of God. But the third and master curse, the Aaron's rod among the serpent causes of distress, is *drink*. While we leave this curse unchecked let us leave the beasts of prey upon our shield, but let us tear the lilies out.

Working Men, Help Yourselves.

Nor can anyone effectually help the working-classes till in these respects they have made a strenuous effort to help themselves. Wherever in any nation there is sloth, incontinence, bad work, recklessness, there is no power on earth which can prevent distress. The working-classes loudly complain that our ships are being filled with foreign sailors, and our trades crowded with foreign competitors; but so it will be if foreign sailors are the less drunken and the more trustworthy, and if the foreign workmen be the more industrious and the less incompetent. That which controls for the good of men the laws of life never will be the shout of the noisiest, the wish of the idlest, the decree of the lowest, but the hand of the diligent and the knowledge of the wise.

We can all Help.

We can all of us help wise institutions—thank God there are many of them. Every effort to make good, steady, diligent workmen, every effort to raise the swarming myriads of our youth into health and purity, into self-reliance and self-respect, every effort to clear our nightly thoroughfares from their shameless impurity, every help given to the well-conducted hospitals, every help energetically and self-denyingly expended on all who are thoroughly willing to help themselves, is a remedy within the power of each of us, and it is a remedy which blesses and is blessed.

The Right Remedy.

Everyone of us belongs to some parish or other, and I am more and more convinced that each parish, as a community, can best cope with distress in its own limits. My own parish lies about Westminster Abbey, and it contains streets as poor as any in London; yet I can answer for my own parish, which, like most others, abounds in thoroughly-planned agencies to raise and comfort the suffering poor—and other clergy will answer for theirs—that is if the laity will help in proportion to their duty, if you, in proportion to your duty, will help in the sort of extemporary collection which you will have an opportunity of giving in your places of worship, then we will undertake that, in some at least of these densely crowded streets not one person, be the coming winter severe as it may, not one person shall suffer cruel and intolerable hardships, unless those hardships be self-induced by drink or crime. The deserving poor are and shall be helped, as they always have been, with clothes, with coals, with work, and that with no grudging hand. Wherever the innocent are suffering need, if each of you will do your individual duty, we shall no longer hear these gloomy and perhaps exaggerated prophecies of the distress of the coming winter. If every man sweeps before his own door, the streets of the New Jerusalem will soon be clean.

